# The Acclimation of *Phaeodactylum tricornutum* to Blue and Red Light Does Not Influence the Photosynthetic Light Reaction but Strongly Disturbs the Carbon Allocation Pattern

**DOI:** 10.1371/journal.pone.0099727

**Published:** 2014-08-11

**Authors:** Anne Jungandreas, Benjamin Schellenberger Costa, Torsten Jakob, Martin von Bergen, Sven Baumann, Christian Wilhelm

**Affiliations:** 1 Department of Plant Physiology, Institute of Biology, Faculty of Biosciences, Pharmacy and Psychology, University of Leipzig, Leipzig, Germany; 2 Department of Computational Landscape Ecology, Helmholtz Centre for Environmental Research - UFZ, Leipzig, Germany; 3 Department of Metabolomics, Helmholtz Centre for Environmental Research - UFZ, Leipzig, Germany; 4 Department of Proteomics, Helmholtz Centre for Environmental Research - UFZ, Leipzig, Germany; 5 Department of Biotechnology, Chemistry and Environmental Engineering, University of Aalborg, Aalborg, Denmark; 6 Institute of Pharmacy, Faculty of Biosciences, Pharmacy and Psychology, University of Leipzig, Leipzig, Germany; University of Hyderabad, India

## Abstract

Diatoms are major contributors to the aquatic primary productivity and show an efficient acclimation ability to changing light intensities. Here, we investigated the acclimation of *Phaeodactylum tricornutum* to different light quality with respect to growth rate, photosynthesis rate, macromolecular composition and the metabolic profile by shifting the light quality from red light (RL) to blue light (BL) and vice versa. Our results show that cultures pre-acclimated to BL and RL exhibited similar growth performance, photosynthesis rates and metabolite profiles. However, light shift experiments revealed rapid and severe changes in the metabolite profile within 15 min as the initial reaction of light acclimation. Thus, during the shift from RL to BL, increased concentrations of amino acids and TCA cycle intermediates were observed whereas during the BL to RL shift the levels of amino acids were decreased and intermediates of glycolysis accumulated. Accordingly, on the time scale of hours the RL to BL shift led to a redirection of carbon into the synthesis of proteins, whereas during the BL to RL shift an accumulation of carbohydrates occurred. Thus, a vast metabolic reorganization of the cells was observed as the initial reaction to changes in light quality. The results are discussed with respect to a putative direct regulation of cellular enzymes by light quality and by transcriptional regulation. Interestingly, the short-term changes in the metabolome were accompanied by changes in the degree of reduction of the plastoquinone pool. Surprisingly, the RL to BL shift led to a severe inhibition of growth within the first 48 h which was not observed during the BL to RL shift. Furthermore, during the phase of growth arrest the photosynthetic performance did not change. We propose arguments that the growth arrest could have been caused by the reorganization of intracellular carbon partitioning.

## Introduction

Diatoms are a highly diverse class of eukaryotic organisms [1] that are widely distributed as phytoplankton species not only in the ocean, but also in freshwater habitats [2]. They have an estimated 40% share of marine primary production [3]. It has been suggested that their ecological success is based on their silica wall, which is formed without assimilated carbon and is therefore less energy demanding than cell walls made from cellulose. A second reason for their ecological success is their capacity to acclimate to dynamic light climates [4] [5]. Diatoms adapt not only to changing light intensities in a very efficient way [6], but also to changes in the light quality. Generally, experiments on the acclimation to light quantity are classified into illumination by photosynthetically non-saturating low light (LL) and saturating high light (HL) intensities. Acclimation to light quality is denoted as the acclimation to a defined range of wavelengths of photosynthetically absorbed radiation. Accordingly, blue light (BL) can be defined as the spectral range with a center wavelength of approximately 460 nm and red light (RL) with a center wavelength of about 660 nm [7].

In diatoms, the light quality was shown to have an effect on chloroplast migration [8], zygote germination [9] and on the light acclimation reactions of photosynthesis [10]–[14]. Recently, it was shown that BL is involved in the light acclimation of *Phaeodactylum tricornutum* [15]. They demonstrated that a typical HL phenotype with a reduced Chl *a* content per cell and a high photosynthetic capacity can be generated only in the presence of BL. Additionally, the photoprotective mechanisms, e.g. a high non-photochemical quenching (NPQ) capacity by means of an active xanthophyll cycle together with the presence of Lhcx proteins [16], can be expressed in the presence of BL, but not under RL. It was shown that the HL or LL phenotype is characterized by a specific gene expression profile that reflects the remodeling of the proteome during the light acclimation process [17].

The molecular background of light acclimation in diatoms is still enigmatic [5]. In green algae, it has been shown that the redox state of the plastoquinone (PQ) pool triggers kinases that regulate not only state 1-state 2 transitions [18], but also the activity of plastidal transcription factors and retrograde signaling [19]. Saturating light intensities cause extensive changes in photosynthetic cells that can be used to indirectly sense illumination, including the redox state of thiols, the thylakoid lumen pH and the accumulation of reactive oxygen species or certain metabolites [20]. Recently, it has been shown that artificial manipulation of the redox state of the PQ pool in *P. tricornutum* by DCMU and DBMIB can alter the gene expression profile according to the pattern expected for light acclimation [21]. However, in diatoms, the PQ pool can become largely reduced during the shift from light to darkness [22]. Moreover, this increased redox state was shown to last even during prolonged dark incubation periods [23]. Therefore, it is questionable how the redox state of the PQ pool could be employed as a trigger of the light flux *in vivo*.

Alternatively to the regulation by the redox state of some components of the photosynthetic apparatus, gene expression could also be regulated by the action of photoreceptors. *P. tricornutum* was shown to possess several blue light-sensing cryptochromes and aureochromes as well as red light sensing phytochromes [5] [24]–[25]. Cryptochromes are a diverse, ubiquitous family of photoreceptors originating from photolyases, which usually have a role in regulating growth and development and serve as circadian clocks [26], while aureochromes are newly described photoreceptors that are restricted to stramenopiles [27] [28]–[29]. The function of the phytochromes is not clear because, in the water column, blue-green light is predominant and red light is strongly attenuated by the water itself. In particular, far red does not penetrate more than 1 m into the water column and phytochromes would not be able to sense the ratio of red light/far red. In this case the switch of the photoreceptor would always be fixed in the “on” position. Therefore, it is likely that blue light sensors are of major importance for light perception. It was shown that PtCPF1 is located in the nucleus and has 6-4 photolyase activity [30]. The aureochromes PtAUREO1a/b and PtAUREO2 are also nucleus located [31] and PtAUREO1a has been shown to act as a transcription factor of dsCYC2, a protein associated with cell cycle progression [32]. Down regulation of the aureo 1a gene also modifies light acclimation [31]. Reduced amounts of aureo1a induce a HL hyper-acclimated state. In contrast to the wild type, transformants with reduced aureo1a concentration were able to acclimate to higher intensities of RL. This was interpreted as a sign that the blue light receptor aureo1a interacts with a RL sensor and might repress HL acclimation in *P. tricornutum* in dependence of the ambient light quality. Putatively, a phytochrome might be such an RL sensor. However, the signaling pathways of these light receptors in diatoms are still largely unknown [5].

Blue light or RL can influence the metabolic network in algae, not only via gene expression and proteome remodeling, but also directly by changing the activity of specific enzymes. BL favors the allocation of carbon into proteins at the expense of carbohydrates in chlorophyta [33]–[34]. It was also shown that in *Chlamydomonas reinhardtii*, BL directly activates the nitrate reductase [35] and has an influence on cell cycle progression [36]. These direct effects should change the metabolome faster than the action via gene expression and subsequent protein biosynthesis, but they should be slower than direct light effects which alter the redox state of components of the photosynthetic primary reactions (reviewed in [37]). Therefore, it can be hypothesized that changes in the redox state of the PQ pool due to differences in the absorption cross sections of the photosystems can be established in the time range of a few minutes. A direct regulation of enzyme activity by light quality will be mirrored in the metabolome in the time range between several minutes to hours, whereas modified protein biosynthesis takes a minimum of half an hour [37].

In this study, we investigated the influence of BL and RL on the metabolic state of cells of *P. tricornutum* on the times scale of minutes to hours and days. For this purpose we compared changes in the metabolome, the macromolecular structure of the cells and the degree of reduction of the PQ pool in semi-continuously grown BL and RL cultures. The light flux and cell numbers were adjusted to an equal photon capture per chlorophyll and time independent of the light quality. Due to the comparison of metabolic changes on different time scales it is possible to hypothesize on the possible mechanisms of regulation of metabolic activity.

## Results

### Pre-acclimated cultures of *P. tricornutum*

Before light shift experiments were started, the cultures of *P. tricornutum* were pre-acclimated to RL and BL conditions for at least 7 days, respectively. All cultures were grown at light/dark cycles of 14/10 h. This photoperiod was kept also during the light quality shift experiments. Importantly, the incident light intensity was adjusted to yield the same amount of absorbed photons per cell and time under both light quality conditions. This resulted in similar growth rates, cellular dry weight and Chl *a* content (Table 1) of RL and BL grown cells of *P. tricornutum*. However, differences were observed in the macromolecular composition of the cells. Typically, BL acclimated cells possessed a significantly higher content of proteins but a decreased concentration of carbohydrates in comparison to RL acclimated cells. This led to a significant difference in the C/N ratio of BL and RL acclimated cells. The lipid content was comparable under both light conditions. These results clearly indicate that BL cells show higher N assimilation activity.

**Table 1:** Growth rate, photosynthetic parameters and the macromolecular composition of BL and RL light acclimated *P. tricornutum* cultures.

	BL	RL	n_BL_	n_RL_	
Growth rate^a^	0.42±0.12	0.42±0.09	15	19	
Chl a^b^	0.58±0.11	0.56±0.08	22	28	
Dry matter^b^	23.9±4.2	21.9±4.5	11	12	
Carbohydrates^c^	30.8±2.5	33.6±3.7	27	28	**
Proteins^c^	44.8±1.7	41.9±2.4	27	28	***
Lipids^c^	14.4±1.8	14.5±1.9	27	28	
C/N^d^	5.9±0.2	6.7±0.1	5	5	***
Φ_PSII_	0.65±0.002	0.67±0.001	6	6	***
O_2_ evolution at growth light^e^	64±9	62±7	10	10	
Maximum O_2_ evolution^e^	238±34	203±31	11	11	*
Maximum non-photochemical quenching	1.05±0.17	0.50±0.12	11	10	***
Xanthophyll cycle pigments^f^	110±3	90±11	4	4	*

Shown are the mean value, standard deviation (±) and the number of replicates (for BL: n_BL_; for RL: n_RL_). The asterisks depict the p-values as described in the method part.

a [d^−1^];

b [pg cell^−1^];

c [% of dry matter];

d [g g^−1^];

e [µmol O_2_ mg Chl a^−1^ h^−1^];

f [mmol mol Chl a^−1^].

Differences between RL and BL acclimated cells were also observed at the level of the primary photosynthetic reaction. The effective quantum yield of PSII (Φ_PSII_) measured at growth light intensity was slightly but significantly lower in cells under BL conditions compared to cells grown under RL (Table 1). This is a surprising result with respect to the fact that gross oxygen evolution rates measured at growth light intensity did not differ between RL and BL conditions (see below). The maximum values of the non-photochemical quenching of Chl *a* fluorescence and the pool size of XC pigments as indicators of the potential of light protection were significantly higher in BL grown cells in comparison to RL grown cells, although the absorbed photon flux density was the same. No diatoxanthin (Dtx) as the second Ddx/Dtx cycle pigment was detected in any of the samples.

### Light shift experiments – long-term acclimation

The light shift experiments of pre-acclimated cultures of *P. tricornutum* from RL to BL and vice versa started 3 h after the beginning of the respective illumination period (t_0_) and were followed over a period of 6 days. Day 0 and t_0_ depict the starting point of the light switch experiments.

**Figure 1:**
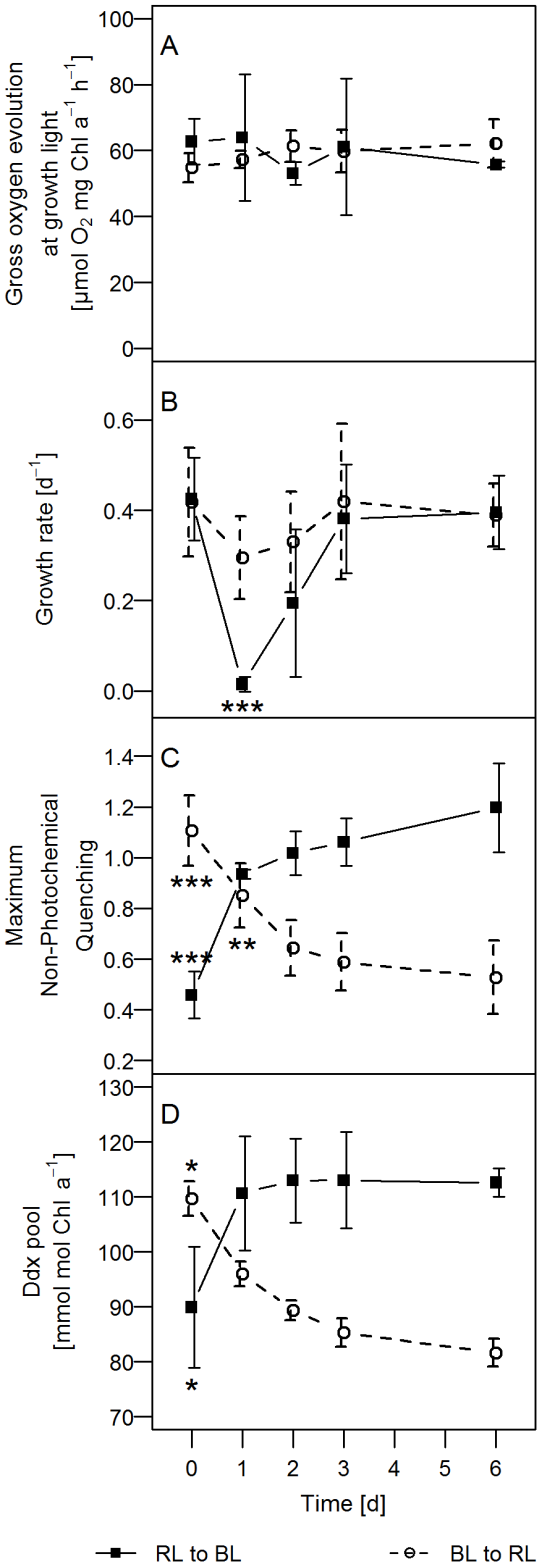
Long-term acclimation to light quality shifts. The acclimation process of *P. tricornutum* cultures was observed for 6 days after the light quality shift from BL to RL and RL to BL. Shown are (A) estimated gross oxygen evolution rates at growth light conditions, (B) growth rate per day, (C) maximum values of non-photochemical quenching and (D) concentration of the xanthophyll cycle pigment Ddx at growth light conditions. The NPQ values were measured at illumination with saturating light intensities, while all other values are measured at growth light conditions. The asterisks depict the p-values of the respective data point compared to the steady state (BL to RL shift is compared to RL steady state, RL to BL shift is compared to BL steady state). n = 4–5. The exact values are specified in Table S1.

No differences were observed in the oxygen-based photosynthesis rates during the entire period of observation (Figure 1 A). Hence, during the light shift experiments all *P. tricornutum* cultures showed comparable gross photosynthesis rates of ∼60 µmol O_2_ mg Chl *a* ^−1^ h^−1^ irrespective of the incident light quality. This stands in contrast to the growth rates that were observed to be slightly reduced during the first 48 h after the light shift from BL to RL (∼0.3 d^−1^ compared to 0.42 d^−1^ in pre-acclimated cultures), whereas in the shift from RL to BL, the growth was completely arrested during the first 24 h of the light shift (Figure 1 B). Growth rates fully recovered to about 0.4 d^−1^ within the next 48 h until day 3 after the start of the light shift. This result was similar when the growth rates were calculated on the basis of the cell number or on the dry matter yield (data not shown).

Within the six days of light shift experiments, the maximal NPQ values and the xanthophyll cycle pool size (Figure 1 C–D) re-adjusted to the values observed in the respective pre-acclimated cultures given in Table 1. In other words, maximum NPQ increased after the shift of RL pre-acclimated cultures to BL from 0.46 (±0.09) to 1.02 (±0.09), a typical value for BL pre-acclimated cultures. The Ddx pool increased from 90 (±11) to 111 (±10) mmol Ddx per mol Chl *a* in the 24 h following the shift and thus reached an amount similar to BL pre-acclimated cultures within just one day. Similarly, the BL to RL shift led to a faster adjustment of the Ddx pool in two days time (from 110±3 to 89±2 mmol Ddx mol Chl *a* ^−1^) compared to the decrease of the NPQ (from 1.12±0.14 to 0.59±0.11) within three days to levels comparable to RL pre-acclimated cultures.

In conclusion, BL cells shifted to a RL acclimated state within three days, as documented by the growth rates, maximal NPQ values and the xanthophyll cycle pool size (Figure 1 B–D). A similar time frame was observed for the switch from RL acclimated cells to BL (Figure 1 B–D). Overall, the largest changes were observed within the first 24 h. This is in accordance with the time course of changes of the macromolecular composition of the cells. The FTIR data revealed that 24 h after the shift from RL to BL and vice versa, the cells resembled the macromolecular composition of cells which were pre-acclimated to similar light conditions (data not shown, compare to Table 1). Thus, in further experiments, we aimed to understand the molecular processes of light acclimation during the first day of the time period of the light shift.

### Fast fluorescence induction kinetics

**Figure 2:**
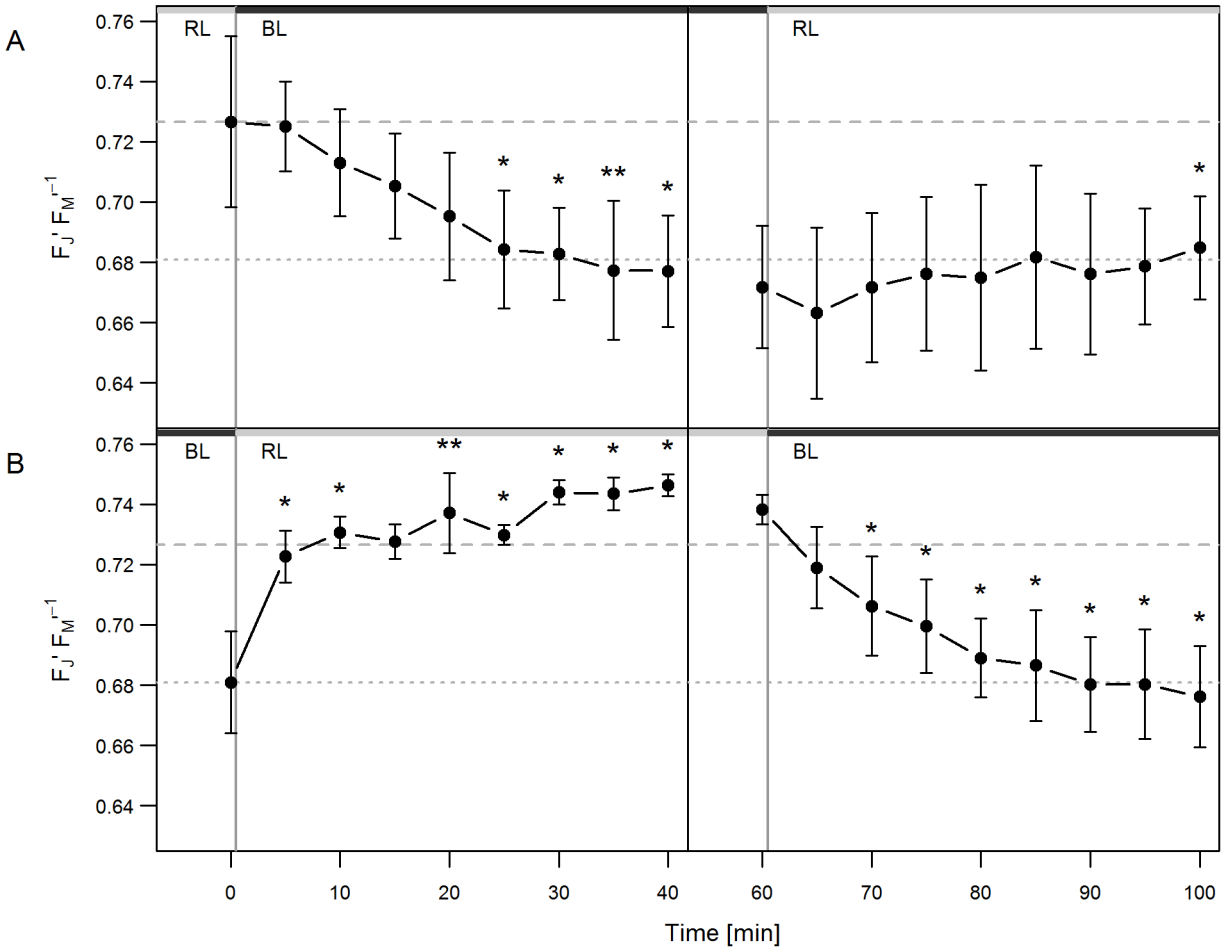
Fast fluorescence induction kinetics. The changes of the J Level of fast fluorescence induction kinetics (F_J_′ F_M_′^−1^) were recorded during the light quality changes. (A) RL to BL shift of a RL adapted culture (0–40 min) followed by a light shift back to RL (60–100 min) (B) BL to RL shift of a BL adapted culture (0–40 min) followed by a light shift back to BL (60–100 min). The horizontal lines show the J level of BL (dotted) and RL (dashed) adapted cultures. The asterisks depict the p-values of the respective data point compared to t_0 min_ (0–40 min) or to t_60 min_ (60–100 min). n = 3. The exact values are specified in Table S2.

Since the photosynthetic redox control is a crucial parameter for light acclimation in higher plants and apparently also in diatoms, changes in the redox state of the PQ pool were characterized. This can be done with the aid of fast fluorescence kinetics of Chl *a* fluorescence [38]. Here, the normalized J level (F_J_′) of the fast fluorescence induction was used as a proxy for the relative redox state of the PQ pool. The higher the ratio F_2 ms_′ F_M_′^−1^ (F_J_′), the more the PQ pool is reduced. This parameter can be measured during the light exposure and therefore reflects the in-situ growth condition. BL acclimated cells showed a slightly lower F_J_′ (and therefore a more oxidized PQ pool) than RL acclimated ones (Figure 2, 0.68 and 0.73 F_J_′ respectively; p = 0.09).

The cells shifted from RL to BL conditions showed a lag phase of 5 min. Then the PQ pool became more oxidized as reflected by a significant decrease of F_J_′, which remained stable after 25 min (Figure 2 A). In contrast, cells that were shifted from BL to RL showed a very fast increase of F_J_′ (Figure 2 B), indicating that the PQ pool becomes immediately more reduced if RL is driving photosynthesis. This change in F_J_′ remained stable after about 30 min. One hour after the light shift, the cells were shifted back to their respective pre-acclimated light condition (see below).

The change in the redox state of the PQ pool is not mainly caused by different activities of PSI and PSII, because BL and RL are absorbed mainly by the FCPs (*fucoxanthin/chlorophyll-binding proteins*) that have identical absorption spectra independent of their PS I or PSII attachment. A second argument that the redox state of the PQ pool is not controlled by the light reactions is the observation that the kinetics are different from BL to RL and from RL to BL. A third argument is supported by Figure 2 (right hand side), which shows that the back reaction from BL to RL is not reversible in contrast to the RL to BL. Finally, the time constant for the redox changes is in the range of half an hour, which would be too slow for changes in the energy distribution between both photosystems. Therefore the observed changes are likely to be induced by alternations in metabolic processes.

### Light shift experiments – short-term acclimation

**Figure 3:**
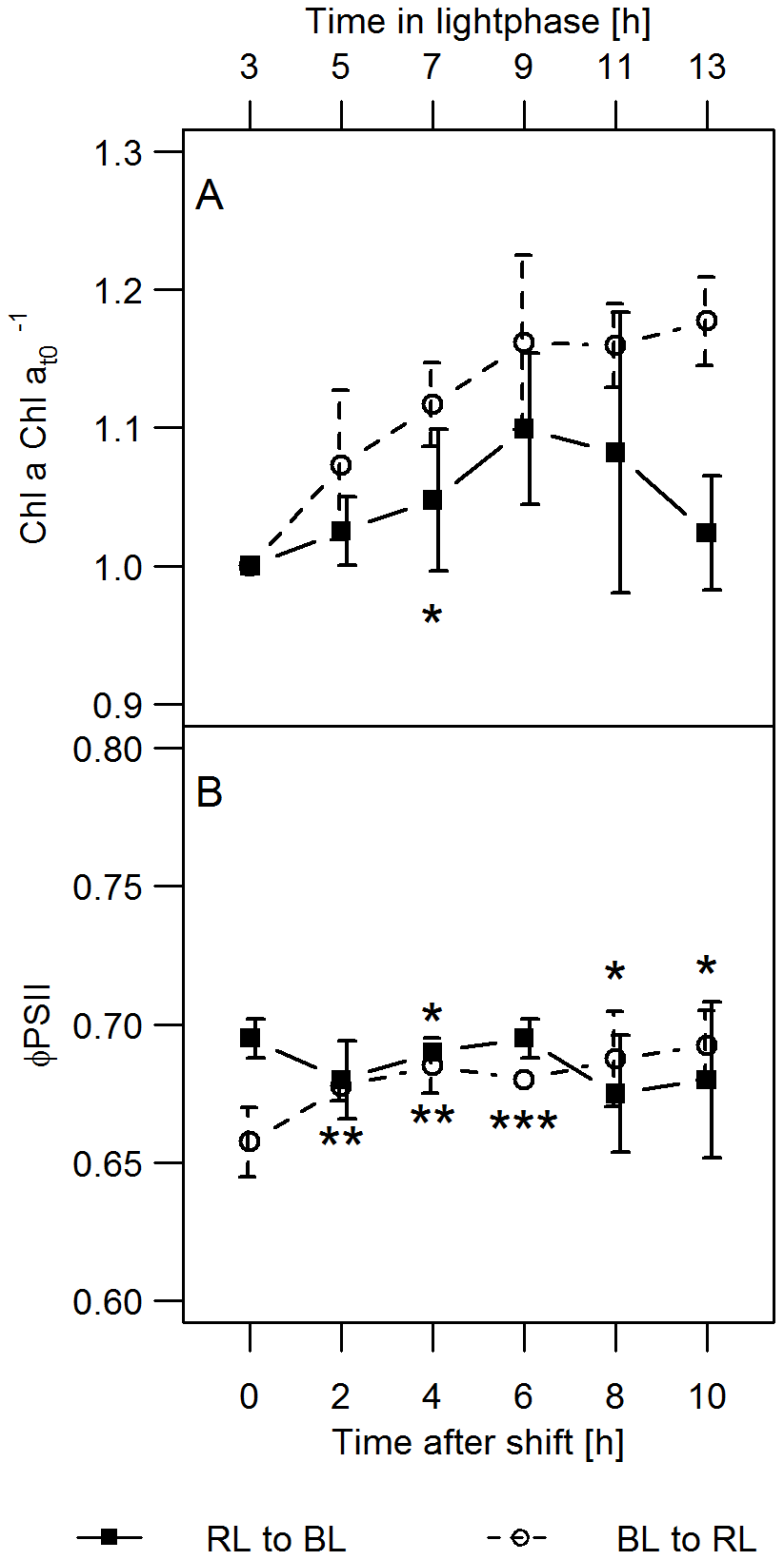
Short-term acclimation to light quality shifts. The changes in (A) the Chl *a* concentration and (B) Φ_PSII_ of *P. tricornutum* cultures were recorded for 10 h after the light quality shift at t_0_ from BL to RL and RL to BL. Additionally, the time in the light phase is given above the plot. The asterisks depict the p-values of the respective data point compared to the steady state the cultures were shifted from at the same time point (Chl *a*) or the daily mean value when this was constant (Φ_PSII_). n = 4–8 for the Chl *a* concentrations and n = 3–4 for F_V_′ F_M_′^−1^. The exact values are specified in Table S3.

After the shift from BL to RL conditions, a 16% increase in the Chl *a* concentration of the algal suspension was observed over a period of 6 h, after which the Chl *a* concentration stagnated until the end of the light period (Figure 3 A). This time course of change of the Chl *a* concentration was also observed in the pre-acclimated cultures under RL and BL, respectively (data not shown). However, after the shift from RL to BL conditions, the increase in Chl *a* concentration was slightly lower during the first 6 h under the new light condition at each sampling point (Figure 3 A). During the next four hours, the Chl *a* concentration of cells shifted to BL decreased again and finally reached the same value as at the starting point of the light shift experiments. This result confirms that growth was arrested in the first 24 h after the RL to BL shift (compare to Figure 1B). In neither experiment did the cell numbers change during the light period (data not shown).

Although cells shifted from RL to BL show impaired Chl *a* biosynthesis and cell division, the effective quantum yield of PSII was only slightly altered and ranged around 0.69 (Figure 3 B), whereas Φ_PSII_ increased slightly from 0.66 (±0.01) to 0.69 (±0.01) in cells that were shifted from BL to RL. At the end of the light period, the same values of Φ_PSII_ were observed in the shifted cultures as in the respective pre-acclimated cultures (Table 1), although Chl *a* synthesis was still lower (BL to RL) or completely blocked (RL to BL) during that time (Figure 1A).

### Changes in the C-partitioning during the light shift experiments

**Figure 4:**
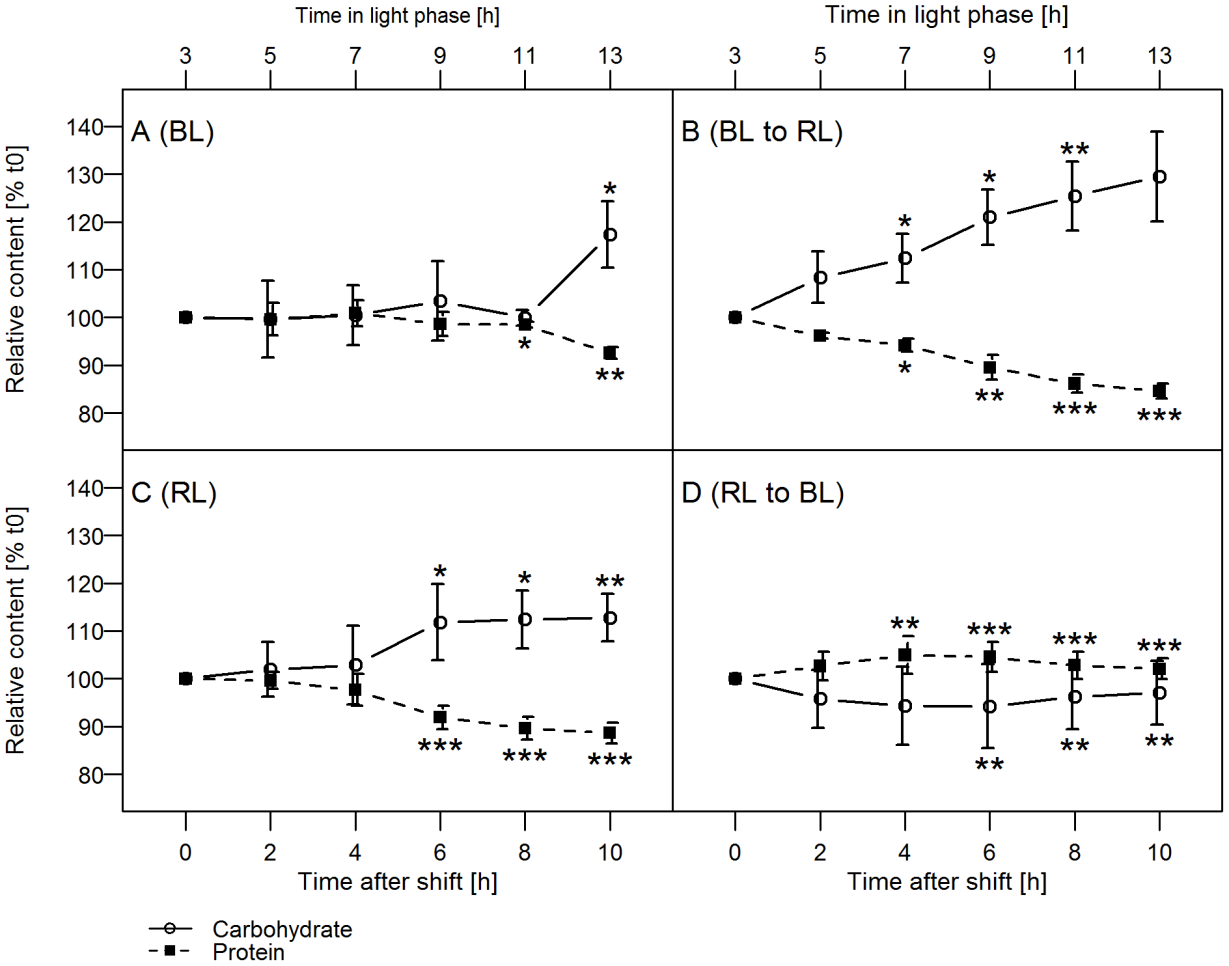
Carbohydrate and protein levels. The carbohydrate and protein levels of *P. tricornutum* cultures during the course of the day were determined for cultures pre-acclimated to (A) BL and (C) RL as well as (B) the changes after a shift from BL to RL and (D) RL to BL. Shown are the relative protein and carbohydrate contents in relation to the values measured at t_0_. The asterisks depict the p-values of the respective data point compared to t_0_ for pre-acclimated cultures (A, C) or compared to the steady state the cultures were shifted from at the same time point (B, D). n = 3–6. The exact values are specified in Table S4.

The analysis of FTIR data allows to follow the changes in the relative pool sizes of carbohydrates, lipids, and proteins. In Figure 4, the relative changes in the macromolecular composition regarding carbohydrates and proteins are presented during the light phase in the shift experiment compared to the controls, which were kept in the same light as before.

In BL pre-acclimated cells the pool sizes of proteins and carbohydrates remained constant, which means that the newly assimilated C is equally directed into the different macromolecular pools during the light phase. Only at the end of the light phase the carbohydrate storage pool increased by 17% at a slight expense of proteins which decreased by 7%. In RL cells, the increase in carbohydrates started earlier in the light phase, but the macromolecular composition remained constant during the first 4 hours. At the end of the light phase, RL and BL pre-acclimated cultures had changed their carbohydrate and protein contents in a comparable manner.

In contrast, directly after the BL to RL shift, the cells strongly increased their pool of carbohydrates at the expense of proteins. The steady decrease of proteins (−15% after 10 h) and increase of carbohydrates (+29% after 10 h) led to significant differences from the BL pre-acclimated cultures already 4 h after the shift. The carbohydrate levels after the shift from BL to RL were also higher (significant at 2/4/8/10 h, not shown in Figure 4) and the protein levels lower (significant at 2/8/10 h, not shown in Figure 4) when compared to the RL pre-acclimated cultures.

**Figure 5:**
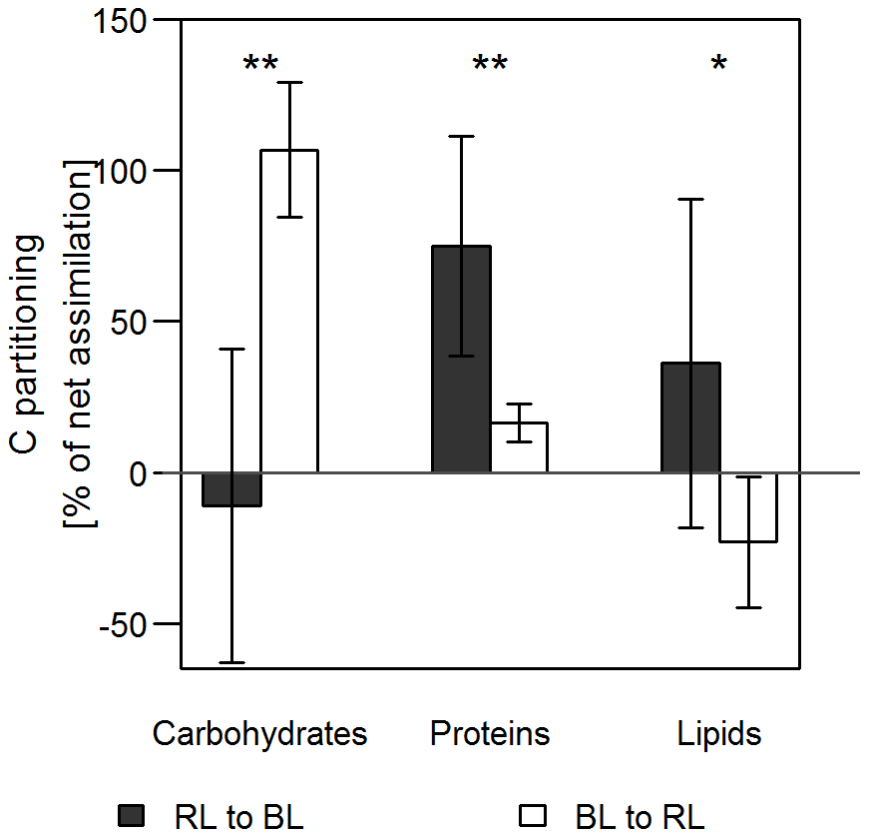
Carbon partitioning. The relative partitioning of C (carbon) into carbohydrates, proteins and lipids was calculated for the 2 h following the light quality changes. Data were calculated on the basis of the FTIR spectra and the net rate of carbon assimilation and, therefore, add up to 100% net carbon assimilation (carbohydrate +protein + lipid bar). Values below zero mean a net loss. Shown are the first 2 h after the light quality change from RL to BL and vice versa. n = 3–6. The exact values are specified in Table S5.

In RL to BL shifted cultures the opposite effect was observed, albeit to a much lower extent. The protein level significantly increased by about 5% within 4 h following the shift to BL (Figure 4 D) in comparison to RL pre-acclimated cultures (Figure 4 C). It should be emphasized that proteins represent the large fraction of macromolecules in *P. tricornutum*. Thus, the small relative increase shown in Figure 4 D equals to a much larger total increase of proteins (see next paragraph and Figure 5). Moreover, the RL to BL was the only experimental condition where a temporary increase of the protein content was observed. The protein pool proved to be increased when compared to the RL pre-acclimated culture (Figure 4 D) and also the BL pre-acclimated culture (significant at 6/8/10 h, not shown in Figure 4).

In addition to the relative changes of macromolecular composition, Figure 5 shows the pattern of carbon allocation into the different pools of macromolecules during the first 2 h of the light shift experiments. Within this time period the total changes of macromolecular composition were very small compared to the pool sizes of macromolecules. This caused a relatively high standard deviation of the calculated carbon allocation rates as shown in Figure 5. Nevertheless, the analysis shows that the carbon partitioning was completely different in the comparison of light shift experiments. Thus, the BL to RL shift was characterized by an almost exclusive flow of newly assimilated carbon into carbohydrates whereas almost no carbon is directed into protein synthesis and a net loss of lipids is observed. The shift from RL to BL showed an opposite pattern with a preferred carbon allocation into proteins and lipids at the expense of carbohydrates.

### Metabolite distribution

This drastic reorganization of the cell internal carbon flux in dependence on changes in the light quality should also be mirrored in the metabolome. Therefore, a metabolome analysis was performed including 36 metabolites that were identified and quantified. In addition to a number of amino acids, the metabolites relate in large part to e.g. glycolysis, the TCA cycle, and the pentose phosphate pathway. No metabolites of the photorespiratory pathway were found in any of the samples.

The relative abundance of metabolites in the cultures pre-acclimated to BL and RL conditions were comparable (Tables S6 and S7). The only significant difference was found in a higher histidine and a lower fructose-6-phosphate content in BL acclimated cells compared to RL acclimated cells. To follow the changes in the metabolite profile of *P. tricornutum*, samples were taken 15 min, 30 min, 1 h and 24 h after the respective shift from one to the other light quality condition.

### Metabolite profile during the shift from RL to BL

**Figure 6:**
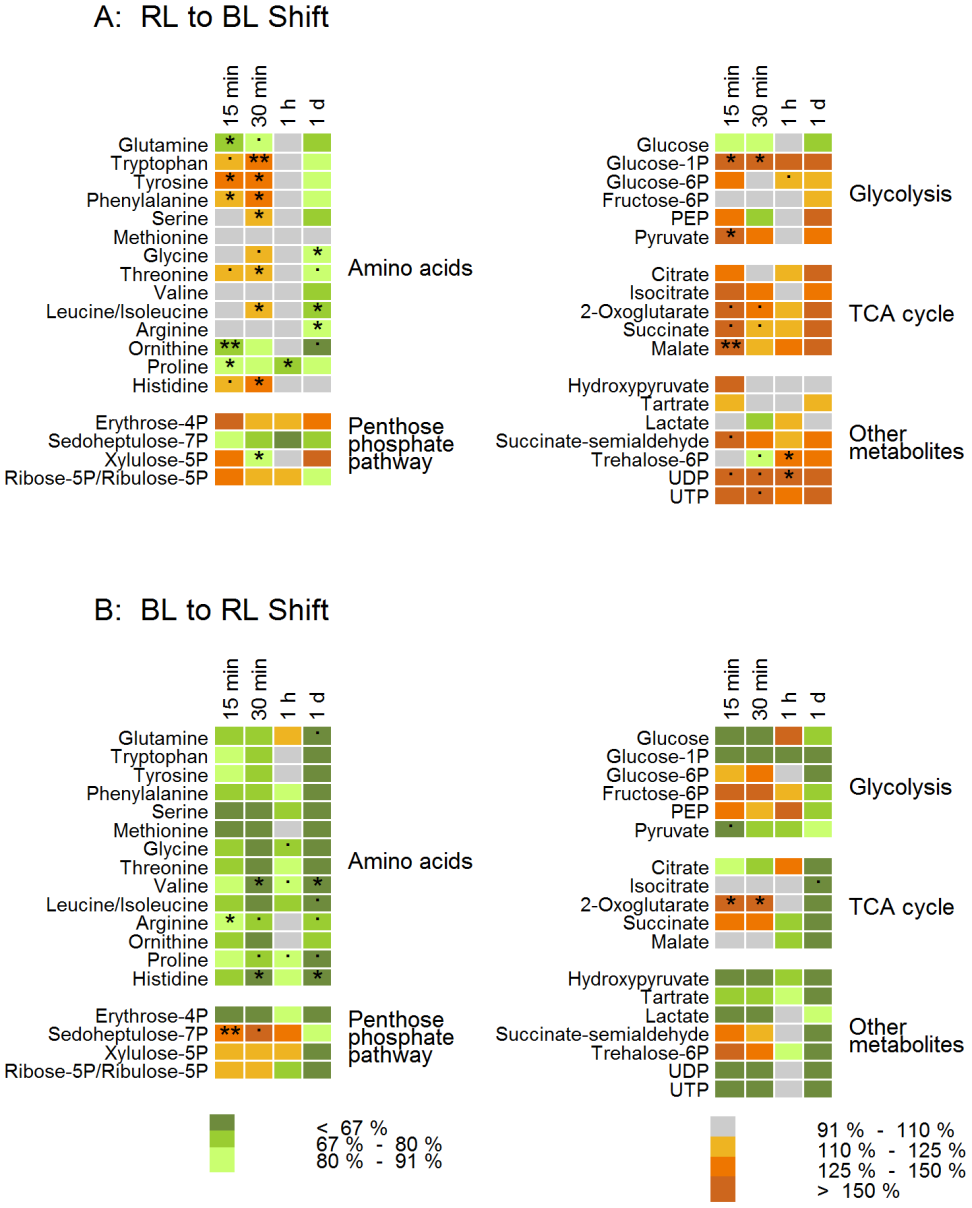
Metabolite profile. The changes in the content of metabolites per cell after a light quality change compared to the pre-acclimated *P. tricornutum* cultures were determined for 4 time points. The asterisks depict the p-values of the respective data point compared to t_0_. Additionally ‘.’ marks p<0.1. Significant changes that were lower than 10% (marked grey) were omitted. n = 3.

In general, for a number of metabolites, a clear increase in their abundance was observed during the first 30 min after the shift from RL to BL conditions. Moreover, already 15 min after the shift to BL conditions, extensive changes in the metabolite profile were detected (Figure 6 A). The most significant changes were found in the pool of amino acids. With the exception of glutamine and ornithine from the urea cycle, the abundance of most of the other amino acids increased. NH_3_ is primarily assimilated into glutamine/glutamate, which represents the precursor for amino acid synthesis [39]. It is therefore likely that the glutamine depletion is due to the increased synthesis of the remaining amino acids. The high levels of most amino acids increased with time (e.g. after 30 min), recovered after 1 h and were found to be lower 24 h after the light shift.

Similarly, a high number of intermediates involved in carbohydrate metabolism were transiently increased after only 15 min BL. This pattern mirrors a transition from an active, carbohydrate-dominated metabolism to a protein biosynthesizing one shortly after the shift. However, the results are not as stringent as for the amino acids. The levels of TCA cycle intermediates clearly increased immediately after the shift to BL. The activated TCA cycle might be explained by an increased need for C skeletons for amino acids. In contrast, apart from an initial increase of glucose-1P and pyruvate, glycolysis intermediate levels were largely unaffected in the first hour. 24 hours after the shift from RL to BL, the relative amount of most metabolites in the central carbon metabolism were increased.

Keeping in mind that in diatoms the final part of the glycolysis is located in the mitochondrion [40], data from the end of these first 24 hours give clear evidence of a strong up-regulation in the reductants in the mitochondrion. This is additionally supported by the high levels of TCA cycle intermediates at the end of this period.

### Metabolite profile during the shift from BL to RL

The changes in the metabolic profile after the shift from BL to RL conditions showed an opposite direction in comparison to the changes observed during the shift from RL to BL conditions. Thus, within 15 to 30 min after the start of the light shift, the abundance of all identified amino acids was decreased (Figure 6 B). Subsequently, the amino acid concentrations approached initial values again, although about half of the amino acid levels were still decreased 1 h after the shift to RL.

In contrast to the switch from RL to BL, there was no consistent response from the TCA cycle intermediates. While 2-oxoglutarate and succinate levels immediately increased after the shift to RL, isocitrate and malate levels remained unchanged. Sedoheptulose-7P was clearly increased shortly after the light switch to RL, while erythrose-4P levels dropped. Therefore, the penthose phosphate pathway also showed a strong initial reaction to RL. The levels of the glycolysis intermediates PEP, fructose-6P and glucose-6P increased while pyruvate levels dropped. One day after the light shift from BL to RL, the amount of all amino acids and of the metabolites related to the central carbon metabolism were clearly decreased.

### Metabolite ratios

**Figure 7:**
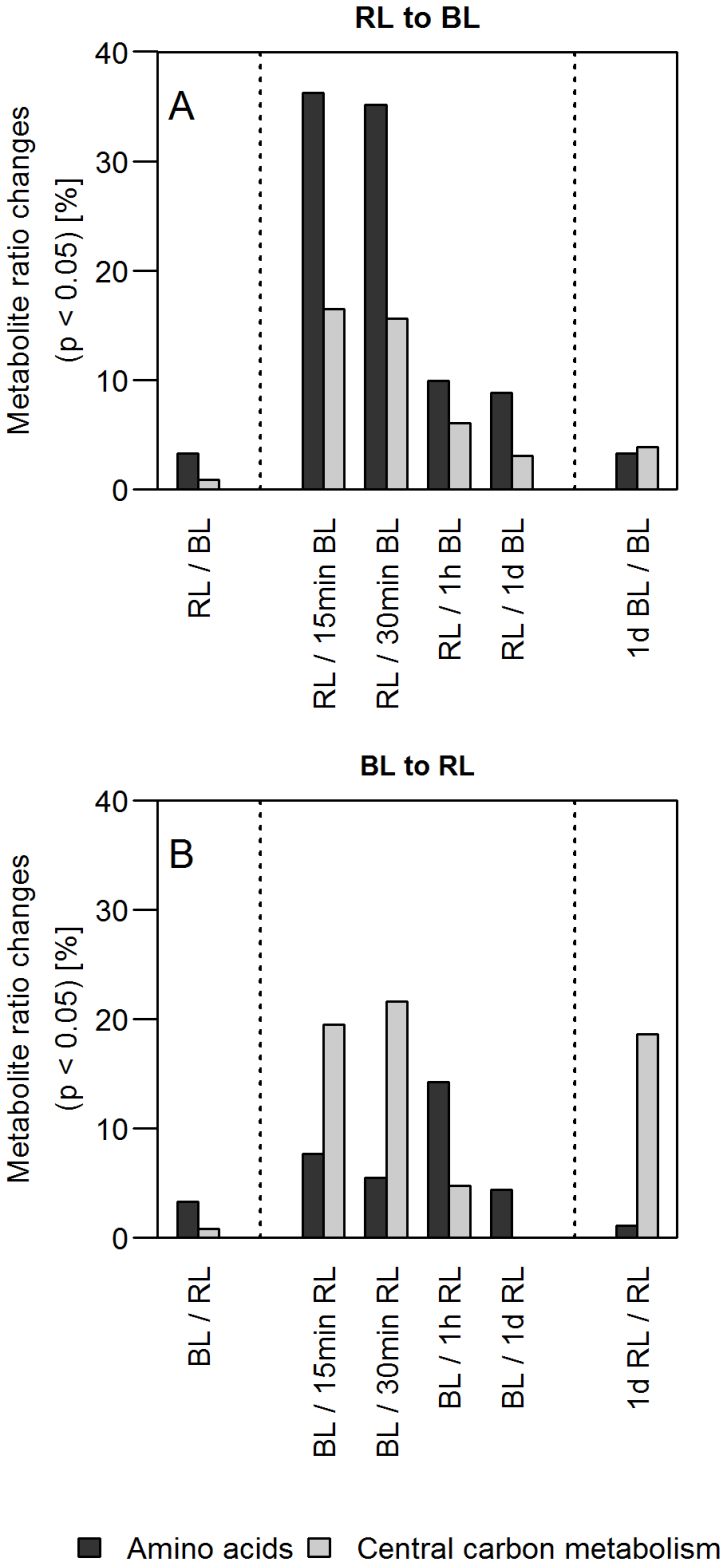
Changes in the metabolic ratios. All possible metabolite ratios (e.g. glucose-6P to fructose-6P) of the measured amino acids (black) or of the metabolites of the central carbon metabolism (grey) were calculated for each time point. The figure shows the rate of significant changes of these ratios between the time points given under each bar (e.g. 10% means that 10% of all metabolite ratios between these time points change significantly, p<0.05). This acts as a value for metabolome reorganization. Given are the relevant time points for the (A) RL to BL shift and (B) the BL to RL shift. The left hand bar is similar for (A) and (B) and compares the RL and BL pre-acclimated cultures. The bars in the middle illustrate the changes between shifted cultures to the light quality they were shifted from. The right hand bar compares the shifted cultures at 1 d after the shift to the light quality they were shifted to.

The metabolite ratios were calculated to visualize the dynamics of the metabolite changes during the early shift phase (Figure 7). A high value means that the ratio of abundance between the different amino acids changes drastically between the time points. This indicates that the production and consumption of the different amino acids are not in equilibrium and the dynamic of metabolome reorganization is high. The value is low under steady state conditions when the flux through the different metabolites is in homeostasis. The same is true for metabolites of the central carbon metabolism.

The metabolite ratios in the pre-acclimated cultures showed very few differences, which confirmed the expectation that an equilibrium in the metabolic pathways is present under both light qualities (left hand side of Figure 7 A, B). During the light shift, the metabolome is strongly disturbed and shows that most significant changes in the metabolome were detected between 15 and 30 min (middle bars in Figure 7 A, B). Moreover, the metabolite ratios confirm that the RL to BL shift induced large changes within the pool of amino acids (Figure 7 A). Interestingly, there was a very fast reorganization in the amino acid pool within the first 15 min and again in the time period from 30 min to 1 h after the start of the light shift. The reorganization between 30 min and 1 h reversed most of the effects that disturbed the metabolism in the first 15 min. The metabolite ratios of the central carbon metabolism were comparable to cells pre-acclimated to BL 24 h after the shift from RL to BL (right hand side of Figure 7 A).

On the other hand, cells shifted from BL to RL conditions showed distinctly less changes in the pool of amino acids (Figure 7 B). Again, the most significant changes occurred in the first 15 min. Although the amount of all amino acids decreased, the overall composition of the amino acid pool stayed quite similar (compare to Figure 6 B). It is noteworthy that 24 h after the shift from BL to RL, cells possessed a significantly different metabolome ratio in comparison to RL pre-acclimated cultures. However, 1 day after the shift to RL no significance was found when compared to the BL pre-acclimated culture, therefore the value is 0 and the respective bar is missing in Figure 7 B.

## Discussion

In the pre-acclimated state, the RL and BL acclimated *P. tricornutum* cultures showed similar values for growth rates, cellular dry matter and Chl *a* content (Table 1) in agreement with previously published results [15]. Since the light conditions were carefully adjusted to the same amount of absorbed radiation under RL and BL conditions, the cells used RL and BL with the same quantum efficiency for growth and showed similar photosynthesis rates.

Despite these similarities, the high light protection via NPQ reached much higher levels in BL pre-acclimated cells. This was already demonstrated by [31], who could show that under non-saturating light conditions, the maximum NPQ seems to be largely determined by the light colour and not by light intensity. The present results suggest that the acclimation to the new light quality is completed after about three days (Figure 1). However, the largest changes occurred within the first 24 h.

Although these light quality induced differences did not change the quantum use efficiency of growth in the pre-acclimated cultures, we observed drastic changes of growth performance during light quality shift experiments (Figure 1 B, Figure 3 A). Whereas within the 48 h following the shift from BL to RL only a small decrease of the growth rate of *P. tricornutum* cells was observed, the shift from RL to BL caused a complete arrest of cell division after 24 h. Growth arrest relaxed within the following 48 h which shows that it represents a temporal imbalance of cell growth. To our knowledge this is the first report of a growth arrest induced by light quality changes.

To explain this surprising observation, a direct, BL induced effect (e.g. photoreceptor-mediated arrest of the cell cycle) or an indirect effect induced by a molecular reorganization of the cells could be proposed. In principle, a direct effect of the BL receptor aureochrome 1a was recently demonstrated in *P. tricornutum* [32]. It was shown that the diatom specific cyclin 2, controlling the G1-to-S phase of the cell cycle, is a transcriptional target of aureochrome 1a. Consequently, a BL triggered transcriptional induction of this cyclin with a subsequent onset of cell cycle was observed. In contrast, under RL the expression of cyclin 2 was inhibited and the onset of cell cycle strongly delayed. The results of [32] stand therefore in contrast to the observation of the present study, where inhibition of the cell division was caused by the shift to BL. The results were, however, obtained by two very different experimental setups. The growth inhibition observed after the shift to BL is temporal (∼2 days), after which the BL acclimated cells show a similar growth rate as the RL acclimated ones. In contrast, the impaired growth reported by [32] is present in acclimated RL cultures of a higher light intensity than it was used in this study (not used by us). Indeed, it was reported elsewhere that, while low BL and RL *P. tricornutum* cultures show identical growth rates, higher RL intensities lead to a reduced growth when compared to BL [31]. The photosynthesis rates remained unaffected during the RL to BL shift and no concomitant increase in cellular dry weight was observed. Therefore, a reduced photosynthesis rate can be excluded as the cause of the impaired growth rate after the shift to BL. An alternative explanation of the observed growth arrest comes into consideration which includes (i) a fast reversal of metabolite distribution and (ii) a macromolecular reorganization of the cells which influences the intracellular carbon partitioning under these conditions.

### Macromolecular reorganization and carbon partitioning

We observed a reorganization of the macromolecular composition of the cells in response to the light quality shifts (Figure 4). Thus, the shift from BL to RL significantly increased the fraction of carbohydrates at the expense of proteins, whereas the RL to BL shift resulted in an increased protein/carbohydrate ratio. The changes in the macromolecular composition yielded in a corresponding increase of the C/N ratio under RL but in a decrease under BL conditions. In accordance with the metabolite distribution pattern the analysis of the partitioning of the newly assimilated carbon by FTIR revealed a drastic increase of carbohydrate synthesis at the expense of protein and lipid synthesis within two hours after the light shift from BL to RL (Figure 5). In contrast, after the shift from RL to BL the synthesis of carbohydrates stagnated whereas protein synthesis was strongly increased. The induction of protein synthesis would also require the activation of the nitrate reductase by BL. Indeed, this activation was demonstrated [30].

The fast changes in the carbon partitioning could be assumed to also have an influence on the redox poise of the chloroplast and the cytosol. This is deduced from the fact that the synthesis of proteins and lipids requires more electrons per carbon than the synthesis of carbohydrates. Thus, after the shift from BL to RL the electron requirement for the preferred synthesis of carbohydrates instead of proteins and lipids could be decreased. As a consequence, it could be assumed that photosynthetically produced reducing equivalents (e.g. NADPH) accumulate. In contrast, after the RL to BL transition the preferred synthesis of proteins at the expense of carbohydrates should consume more electrons and should drain the pool of reducing equivalents. Support for this hypothesis comes from the changes of the redox state of PQ pool in *P. tricornutum* during light shift experiments (Figure 2). There are a number of studies that show the increase of the degree of reduction of the PQ pool by stromal reductants in diatoms [4] [41] but also in green algae [42]. In accordance with the assumptions stated above, in the present study the observed changes in the J level of fast fluorescence induction correlate with an increased degree of reduction of PQ after the BL to RL shift whereas the PQ pool was less reduced after the RL to BL shift. Since the photosynthesis rates at growth light intensity did not change during the light shift experiments it could be hypothesized that the changes in the degree of reduction of the PQ pool are not primarily under control of the energy balance between the photosystems or the balance between light and dark reactions but can be regarded as a effect of metabolic redox control by the changes in carbon partitioning and of the macromolecular composition of the cells. Such a new model of redox control needs further confirmation by time resolved gene expression studies compared to changes in the proteome and metabolome.

In *P. tricornutum*, the RL to BL shift and the accompanied induction of protein synthesis could be a probable reason for the observed growth arrest as outlined in the following hypothesis. Diatoms are known for their ability to reduce nitrate in excess of their metabolic demand. It was suggested that the subsequent release of reduced nitrogen as ammonium into the surrounding medium functions to maintain the energy balance within the cell [43]. The increased demand of carbon skeletons for protein biosynthesis during the shift from RL to BL might reduce the amount of cellular carbon available for basic metabolism which, in combination with an excretion of reduced nitrogen, could be the reason why the cell growth stopped until this imbalance was readjusted. The inverse shift from BL to RL did not initiate such an enhanced N reduction and the acclimation was performed by a continuous metabolic reorganization. This new hypothesis presented could be tested in a future study by a data set obtained from similar experiments when *P. tricornutum* is grown with ammonium instead of nitrate as the source of nitrogen. Ammonium is known to inactivate the nitrate reductase in diatoms [44]. Thus, if the activation of the nitrate reductase is responsible for the observed physiological effects during the shift from RL to BL conditions, this acclimation pattern should not be present under cultivation with ammonium.

### Fast reversal of metabolite distribution during light quality shifts

The light shift experiments revealed a significant reversal of the distribution of cellular metabolites (Figure 6, Figure 7). Importantly, the initial kinetics of the changes of metabolites was in the range of minutes. To our knowledge, this should be faster than it could be expected due to an altered protein profile as a consequence of gene expression. The time frame given for the effects of transcriptional changes is about 30 min [37]. Although we cannot exclude the interference of a fast control by gene expression, the fast changes of the metabolite levels raise the hypothesis that some of the crucial enzymes are under direct regulatory control by light quality. Indeed, in green algae similar changes in the macromolecular composition of the cells were observed in a comparable time frame during light shift experiments with BL and RL [33] [45]. Crude cell extracts showed light quality dependent changes in the turnover rate of enzymes like e.g. amylase, aldolase, pyruvate kinase, and NAD-dependent isocitrate dehydrogenase [33]. In particular, for pyruvate kinase (PK) large changes in activity were observed. This enzyme is a regulatory site of glycolysis and catalyses the formation of pyruvate from phosphoenolpyruvate (PEP) which is then the precursor of the TCA cycle and thus, also for the synthesis of amino acids. Blue light strongly enhances the turnover rate of PK [33] and should promote the formation of pyruvate. In the present study a corresponding significant increase of the level of pyruvate was detected after the shift from RL to BL. In addition, a clear increase of the metabolites of the TCA cycle suggests an up-regulated activity of this cycle. A high activity of the TCA cycle is required for the synthesis of a number of amino acids and is thus, in accordance with the observed increase in the protein pool.

In contrast, after a BL to RL shift a decrease in the pyruvate/PEP ratio and the accumulation of glycolytic metabolites upstream of pyruvate indicate a lower activity of PK and thus a down-regulation of the supply of pyruvate through glycolysis. Consistently, the decreased formation of pyruvate could be correlated with the lower level of citrate, the initial metabolite in the TCA cycle. Thus, a down regulation of sugar degradation by glycolysis and a reduced TCA cycle activity should result in an accumulation of carbohydrates and in a reduced synthesis of amino acids and proteins. Indeed, both were observed in the analysis of macromolecular composition of cells during the BL to RL shift experiments.

The RL to BL shift led to a clear decrease of the glutamine concentration. Correspondingly, the levels of about half of the other measured amino acids increased. It is known that glutamate/glutamine acts as the precursor for amino acid synthesis [39]. However, chlorophylls are also derived from glutamic acid. It is therefore possible that the decreased level of this precursor due to the enhanced amino acid synthesis is responsible for the initial suppress of Chl *a* synthesis.

The exact mode of action of the activity regulation of metabolic enzymes by light quality is not resolved up to now. It should be kept in mind that the proposed regulation of enzymes by light quality was not yet shown for *P. tricornutum*, and should be confirmed in further studies. Importantly, the metabolic changes take place outside of the chloroplast and were observed also in non-green cells [33]. Therefore, the regulation of enzyme activity can be assumed to be independent of the photosynthetic reaction. Instead, it was suggested that a direct regulation of enzyme activity could occur by BL absorbing components, such as flavins like FMN (*flavin mononucleotide*) or FAD (*flavin adenine dinucleotide*). Interestingly, such BL induced activation is known for FAD-containing enzymes, e.g. pyruvate dehydrogenase and nitrate reductase [35]. In contrast, FMN-containing enzymes seem to be inactivated by BL [45]. It is important to note that this mode of enzyme regulation is relevant for the observed changes in the metabolome on the time scale of minutes. On the other hand, it is reasonable to assume that the sustained changes of the macromolecular composition of the cells after light shift experiments were also regulated by *de novo* synthesis of enzymes. Here, in particular BL can be assumed to act on the regulation of transcription and translation of genetic information. This conclusion was deduced from the observation that the effects of a short-term illumination with BL do not disappear immediately after the transfer back to RL illumination [45]. In the present study a similar effect was observed on the changes of the degree of reduction of the PQ pool during shift experiments from RL to BL and back to RL.

## Conclusions

In conclusion we did not find a satisfactory explanation for the observed growth arrest in light shift experiments from RL to BL. However, we can exclude an inhibitory effect by the limitation of the photosynthetic reaction. Moreover, a remarkable result of the present study is the finding that the light acclimation process in *P. tricornutum* is characterized by the disturbance of the cellular carbon allocation revealed by a metabolic reorganization of the cells as a first response to the new light environment. It is hypothesized that this fast initial reaction of the cells in response to light quality changes could be assigned to a direct control of enzyme activity by BL and RL. The second phase of metabolic changes in the range of 30 min to 1 hour would subsequently represent the effects of transcriptional changes [37]. Further studies on gene expression and protein levels need to be done to clear up the effects ascribed to a direct enzyme regulation and transcriptional control via photoreceptors in *P. tricornutum*.

The initial reorganization of the metabolite profile was accompanied by changes in the degree of reduction of the PQ pool. We propose that these changes were not directly due to shifts in light quality. Instead it could be hypothesized that the degree of reduction of the PQ pool is also under metabolic control due to changes in the cellular carbon partitioning.

Finally, we hypothesize that photoreceptors could fulfill both functions: the regulation of the activity of key enzymes of N and C assimilation and the regulation of gene transcription. The proposed participation of photoreceptors on the regulation of cellular carbon allocation will be a subject of future research.

## Materials and Methods

### Growth conditions

*P. tricornutum* UTEX 646 was grown as previously described in [15] in modified f/2 medium [46] with 16 g l^−1^ marine salt concentration and no silica. The cells were grown semi-continuously as airlift-cultures in a rectangular culture tank of 3 cm depth under illumination with either blue (465±10 nm) or red (660±10 nm) at light intensities of 24 and 40 µmol photons m^−2^ s^−1^, respectively. This specific intensity of irradiance with BL and RL resulted in a similar amount of photosynthetically absorbed radiation (Q_Phar_) of 10 µmol photons m^−2^ s^−1^ (calculated as described in [47]). The illumination was provided by Flora LED-panels (CLF Plant Climatics, Wertingen, Germany) or by self-manufactured LED-panels with 14/10 h light/dark cycles. The cultures were diluted daily to achieve a chlorophyll (Chl) *a* concentration of 2 mg l^−1^ at 3 h into the light phase. All experiments were carried out with cultures which were acclimated at least 7 days to the described conditions.

Daily samples for the determination of cell numbers, pigment content and dry matter (DM) as well as FTIR (*fourier-transform infrared*) spectra were always taken 3 h after the start of the light phase. Light shift experiments were also started at this time point.

For the light shift experiments, the illumination of the pre-acclimated cultures was changed from RL to BL or vice versa. Samples for the determination of Chl *a* concentration, effective quantum efficiency (see below), cell numbers and FTIR spectra were taken every 2 h for a period of 10 h after the light quality change to evaluate fast changes during the acclimation process. Additionally, the daily development of these cell characteristics was measured in pre-acclimated RL and BL cultures for the same time points as a reference.

The long-term acclimation process was evaluated by sampling at days 1, 2, 3 and 6 after the light quality change. At these time points, additional measurements were carried out to determine the oxygen-based photosynthetic rates, non-photochemical quenching and diadinoxanthin (Ddx) concentrations.

### Growth, pigmentation and photosynthetic parameters

Cell numbers were determined with a particle counter (Z2 Beckman Coulter GmbH, Krefeld, Germany). Dry matter determination was carried out by harvesting 30–50 ml of the culture by centrifugation (3000 *g, 5 min). The pellets were washed twice with distilled water, frozen in liquid nitrogen, freeze-dried (Labconco FreeZone, ILMVAC GmbH, Ilmenau, Germany) and weighted. To calculate the dry matter per cell, the cell number of the sample was counted prior to the last centrifugation step.

The growth rate was calculated as gain per day based on Chl *a* (growth rate [d^−1^] = Chl *a*_d1_ Chl *a*_d0_^−1^−1). Therefore, a doubling of the Chl *a* content would result in a growth rate of 1, corresponding to an increase of 100%.

For pigment analysis cells were harvested on glass-fiber filters and freeze-dried. Subsequently, the cells were broken with glass beads using a cell homogenizer (Braun, Melsungen, Germany) as explained in [6]. Chl *a* concentration was determined by extraction in 90% acetone according to [48]. Ddx concentration was determined by HPLC (*high pressure liquid chromatography*) using a Waters 600 MS system following the method described in [49].

Oxygen-based photosynthetic rates were measured with a Clark-type electrode (MI730, Microelectrodes Inc., Bedford, NH, USA) at light intensities between 0 and 1500 µmol photons m^−2^ s^−1^ as described in detail in [6]. Simultaneously, fluorescence was detected by a PAM (*pulse amplitude modulation*) fluorometer (PAM 101/103, Walz Effeltrich, Germany). The NPQ was calculated based on the fluorescence as described in [50] at the highest measured light intensity (∼1500 µmol photons m^−2^ s^−1^).

A Handy PEA (Handy PEA with Liquid Phase Adapter, Hansatech Instruments LTD, UK) was used to record fast fluorescence induction kinetics of algal samples taken directly from the cultures. The so-called OJIP transients depict the course of the fluorescence rise from minimum (F_O_) to maximum (F_M_) levels when a sample is illuminated with saturating light. Different fluorescence levels become visible that are called J (at 2 ms), I (at 30 ms) and P (at 220 ms) levels by logarithmic scaling [51]. The normalized value of the J level (F_J_; F_J_ = F_2 ms_ F_M_^−1^) correlates with the redox state of the plastoquinone pool in the thylakoid membrane [38]. To record the OJIP transients of our cultures, light-adapted samples were directly illuminated with 3500 µmol photons m^−2^ s^−1^ for 10 seconds for fluorescence measurement. As the signal was recorded in pre-illuminated cells, all fluorescence designations are marked by a prime (e.g. F_M_′). OJIP transients were recorded every 5 min for a period of 40 min after the light quality shift. After 1 h, the cultures were shifted back to their original light quality. Again, the OJIP transients were recorded every 5 min for a period of 40 min. The minimum and maximum values of the measured OJIP transients were also used for the calculation of the effective quantum yield of PSII (Φ_PSII_ = (F_M_′−F_O_′) F_M_^−1^′).

### Macromolecular cell composition via FTIR spectroscopy

The macromolecular composition of a dried algae sample was determined via its FTIR spectrum [52]. FTIR spectroscopy is based on the absorption of chemical groups (e.g. C = O) at individual wave numbers in the mid-infrared range. As the absorption pattern of given molecules is distinct, this method is able to identify substances contained in a measured sample. Accordingly it can be used to quantify the amount of carbohydrates, proteins and lipids [52] or other substances [53] in small algae cell samples. Since light driven changes in the macromolecular composition of the cells can be observed already after 30 min, FTIR can follow the distribution of the newly synthesized carbon or cell internal carbon recycling in the time range of hours. For this purpose, the cells were centrifuged twice at 5000 *g for 3 min and re-suspended in distilled water to wash and concentrate them. For each sample, 6 replicates with a volume of 2 µl were placed on a microtiter plate and dried at 40°C. The FTIR spectra were recorded in the range of 4000–700 cm^−1^ with a resolution of 4 cm^−1^ and 32 scans per spot (HTS-XT microtiter plate module and Vector 22 laser unit, Bruker Optics, Germany) [52]. The recorded spectra were processed by using a Blackman-Harris apodization and excluding the CO_2_ absorption band, followed by a baseline correction (rubber band method with 64 points).

Spectra of laminarine (carbohydrate reference), glycerol tripalmitate (lipid reference) and bovine serum albumin (protein reference) were used as reference spectra to determine the abundance of carbohydrates, lipids and proteins in the recorded spectra by a PLS (partial least square) regression in the range of 1800–900 cm^−1^. These three substance classes were assumed to represent 100% of the cellular dry matter.

The carbon (C) and nitrogen (N) content was determined with a CHN elemental analyzer (vario EL, Analytik Jena GmbH) using the dried samples after dry matter measurement.

### Calculation of C-partitioning

The rates of net assimilated carbon were calculated based on the measured photosynthetic rates (based on oxygen evolution), dry weight and the cell composition (based on the FTIR spectroscopy results). The amount of C per dry matter and the required electrons per biomass for carbohydrates, proteins and lipids were included as specified in [54]. For a calculation example, see File S1.

### Metabolomics

To get an overview of the metabolites of the core metabolic pathways and amino acid concentrations, samples were taken directly prior as well as 15 min, 30 min, 1 h and 24 h after the light quality shift. For each time point, three replicate samples were taken, extracted and analyzed. In addition, the experiments were independently repeated three times.

About 3*10^7^ cells (∼0.6 mg DM) were filtered on a glass fiber filter, washed with distilled water, frozen in liquid nitrogen, freeze-dried and stored at −80°C. The sampling procedure took 20–25 seconds until freezing. The extraction was carried out in PVC reaction tubes. Glass beads, 1 µg ^13^C glucose (used as standard for quantification of all measured metabolites) and 2.4 ml methanol were added to the filter. The cells on the filter were homogenized in a Precellys Homogenisator (2×20 sec * 6500 *g). Distilled water (0.6 ml) was added after the extraction. The samples were centrifuged twice at 21000 *g for 1 min to remove the remaining filter pieces, glass beads and bigger cell pieces and frozen again at −80°C until analysis.

Amino acid concentrations from cell lysates were determined using a targeted metabolic approach with the AbsoluteIDQ p150 kit (BIOCRATES Life Sciences AG, Innsbruck, Austria) as described earlier [55]. Briefly, 30 µl of supernatants were prepared according the manufacturer's protocol. FIA-MS/MS analyses were carried out on an Agilent 1100 series binary HPLC system (Agilent Technologies, Waldbronn, Germany) coupled to an 4000 QTrap mass spectrometer (AB Sciex, Concord, Canada) equipped with a TurboIon spray source. Quantification was achieved by positive and negative multiple reaction monitoring (MRM) detection in combination with the use of stable isotope-labeled and other internal standards. Data evaluation for quantification of metabolite concentrations was performed with the MetIQ software package.

For ion chromatography-tandem mass spectrometry (IC-MS/MS)-based analysis extracts (25 µl) were analyzed on an ICS-5000 (Thermo Fisher Scientific, Dreieich, Germany) coupled to an API 5500 QTrap (AB Sciex). Separation was achieved on an IonPac AS11-HC column (2×250 mm, Thermo Fisher Scientific) with an increasing potassium hydroxide gradient. MS analysis was performed in MRM mode using negative electrospray ionization and included organic acids, carbohydrates and nucleotides involved in central metabolite pathways.

### Statistics

The statistical analysis was carried out using R 2.15.2 and RStudio 0.96.331 (R Core Team 2012). The Student's *t*-test was used for the comparison of two data sets. Data sets were considered paired if the samples were taken from the same culture in a time frame of less than 24 hours. The p-values are marked with * (p≤0.05), ** ((p≤0.01) and *** (p≤0.001). The amount of replicates (n) for each experiment is specified in the figure legends.

## Supporting Information

Table S1 **Long-term acclimation to light quality shifts.** The gross oxygen evolution, growth rates, maximum NPQ and Ddx pool were followed for 6 days after the RL to BL and the BL to RL shifts.
(PDF)

Table S2 **Fast fluorescence induction kinetics.** The changes of the J Level of fast fluorescence induction kinetics (F_J_′ F_M_′^−1^) were recorded during the light quality changes. after 60 min, the shift was reversed.
(PDF)

Table S3 **Short-term acclimation to light quality shifts.** The changes in the Chl *a* concentration and Φ_PSII_ of *P. tricornutum* cultures were recorded for 10 h after the light quality shift.
(PDF)

Table S4 **Carbohydrate and protein levels.** The changes in the carbohydrate and protein levels of *P. tricornutum* cultures were recorded for 10 h after the light quality shift and in the respective BL and RL pre-acclimated reference cultures.
(PDF)

Table S5 **Carbon partitioning.** The relative partitioning of C (carbon) into carbohydrates, proteins and lipids was calculated for the 2 h following the light quality changes.
(PDF)

Table S6 **Relative metabolite concentrations in BL and RL pre-acclimated** ***P. tricurnutum*** cultures (in relative counts * cell^−1^). Significant differences were calculated by a Student's t-test.
(PDF)

Table S7 **Amino acid concentrations in BL and RL pre-acclimated** ***P. tricurnutum*** cultures (in 10^−3^ pmol * cell^−1^). Significant differences were calculated by a Student's t-test.
(PDF)

File S1 **The calculation of net C partitioning into biomass for carbohydrates, proteins and lipids.** A calculation example is given at the right hand side.
(PDF)
