# Supplementary material for: The Acclimation of Phaeodactylum tricornutum to Blue and Red Light Does Not Influence the Photosynthetic Light Reaction but Strongly Disturbs the Carbon Allocation Pattern
Source: PLoS One. 2014 Aug 11;9(8):e99727. doi: 10.1371/journal.pone.0099727 (PMC4128583; doi:10.1371/journal.pone.0099727)
Supplement: Table S1 — Long-term acclimation to light quality shifts. The gross oxygen evolution, growth rates, maximum NPQ and Ddx pool were followed for 6 days after the RL to BL and the BL to RL shifts. (PDF) [file pone.0099727.s001.pdf]

Table S1: Long-term acclimation to light quality shifts. The gross oxygen evolution, growth rates, maximum NPQ and Ddx pool were followed for 6 days after the RL to BL and the BL to RL shifts.

Day	0	1	2	3	6
**RL to BL shift**					
gross O_2 evolution at growth light [µmol O_2 mg Chl a^{-1} h^{-1}]	63 ± 7	64 ± 19	53 ± 3	61 ± 21	56 ± 1
Growth rate [d^{-1}]	0.42 ± 0.09	0.01 ± 0.02	0.19 ± 0.16	0.38 ± 0.12	0 .40 ±0.08
Maximum NPQ	0.46 ± 0.09	0.94 ± 0.02	1.02 ± 0.09	1.06 ± 0.09	1.20 ± 0.18
Ddx pool [mmol mol Chl a^{-1}]	90 ± 11	111 ± 10	113 ± 8	113 ± 9	113 ± 3
**BL to RL shift**					
gross O_2 evolution at growth light [µmol O_2 mg Chl a^{-1} h^{-1}]	55 ± 4	57 ± 3	61 ± 5	60 ± 6	62 ± 7
Growth rate [d^{-1}]	0.42 ± 0.12	0.30 ± 0.09	0.33 ± 0.11	0.42 ± 0.17	0 .39 ± 0.07
Maximum NPQ	1.12 ± 0.14	0.85 ± 0.13	0.64 ± 0.11	0.59 ± 0.11	0.53 ± 0.15
Ddx pool [mmol mol Chl a^{-1}]	110 ± 3	96 ± 2	89 ± 2	85 ± 3	82 ± 3
