# Supplementary material for: The Acclimation of Phaeodactylum tricornutum to Blue and Red Light Does Not Influence the Photosynthetic Light Reaction but Strongly Disturbs the Carbon Allocation Pattern
Source: PLoS One. 2014 Aug 11;9(8):e99727. doi: 10.1371/journal.pone.0099727 (PMC4128583; doi:10.1371/journal.pone.0099727)
Supplement: Table S2 — Fast fluorescence induction kinetics. The changes of the J Level of fast fluorescence induction kinetics (FJ′ FM′−1) were recorded during the light quality changes. after 60 min, the shift was reversed. (PDF) [file pone.0099727.s002.pdf]

Table S2: Fast fluorescence induction kinetics. The changes of the J Level of fast fluorescence induction kinetics (F_J' F_M'$^{-1}$) were recorded during the light quality changes. after 60 min, the shift was reversed.

**Time [min]**	**0**	**5**	**10**	**15**	**20**	**25**	**30**	**35**	**40**
RL to BL Shift	0.727 ± 0.028	0.725 ± 0.015	0.713 ± 0.018	0.705 ± 0.017	0.695 ± 0.021	0.684 ± 0.020	0.683 ± 0.015	0.677 ± 0.023	0.677 ± 0.019
**Time [min]**	**60**	**65**	**70**	**75**	**80**	**85**	**90**	**95**	**100**
Backshift to RL	0.672 ± 0.020	0.663 ± 0.028	0.672 ± 0.024	0.676 ± 0.026	0.675 ± 0.031	0.682 ± 0.030	0.676 ± 0.027	0.679 ± 0.019	0.685 ± 0.017
**Time [min]**	**0**	**5**	**10**	**15**	**20**	**25**	**30**	**35**	**40**
BL to RL Shift	0.681 ± 0.017	0.723 ± 0.009	0.731 ± 0.005	0.728 ± 0.006	0.737 ±0.013	0.730 ± 0.003	0.744 ± 0.004	0.744 ± 0.006	0.746 ± 0.004
**Time [min]**	**60**	**65**	**70**	**75**	**80**	**85**	**90**	**95**	**100**
Backshift to BL	0.738 ± 0.005	0.719 ±0.013	0.706 ± 0.017	0.699 ± 0.016	0.689 ± 0.013	0.686 ± 0.018	0.680 ± 0.016	0.680 ± 0.018	0.676 ± 0.017
