# Supplementary material for: The Acclimation of Phaeodactylum tricornutum to Blue and Red Light Does Not Influence the Photosynthetic Light Reaction but Strongly Disturbs the Carbon Allocation Pattern
Source: PLoS One. 2014 Aug 11;9(8):e99727. doi: 10.1371/journal.pone.0099727 (PMC4128583; doi:10.1371/journal.pone.0099727)
Supplement: Table S3 — Short-term acclimation to light quality shifts. The changes in the Chl a concentration and ΦPSII of P. tricornutum cultures were recorded for 10 h after the light quality shift. (PDF) [file pone.0099727.s003.pdf]

Table S3: Short-term acclimation to light quality shifts. The changes in the Chl *a* concentration and Φ_{PSII} of *P. tricornutum* cultures were recorded for 10 h after the light quality shift.

**Time after t_0 (h)**	**0**	**2**	**4**	**6**	**8**	**10**
**RL to BL Shift**						
Chl *a* [%]	1	1.03 ± 0.02	1.05 ± 0.05	1.10 ± 0.05	1.08 ± 0.10	1.02 ± 0.04
$F_v\ F_m^{-1}$	0.70 ± 0.01	0.68 ± 0.01	0.69 ± 0.00	0.70 ± 0.01	0.68 ± 0.02	0.68 ± 0.03
**BL to RL Shift**						
Chl *a* [%]	1	1.07 ± 0.05	1.12 ± 0.03	1.16 ± 0.06	1.16 ± 0.03	1.18 ± 0.03
$F_v\ F_m^{-1}$	0.66 ± 0.01	0.68 ± 0.01	0.69 ± 0.01	0.68 ± 0.00	0.69 ± 0.02	0.69 ± 0.01
