# Supplementary material for: The Acclimation of Phaeodactylum tricornutum to Blue and Red Light Does Not Influence the Photosynthetic Light Reaction but Strongly Disturbs the Carbon Allocation Pattern
Source: PLoS One. 2014 Aug 11;9(8):e99727. doi: 10.1371/journal.pone.0099727 (PMC4128583; doi:10.1371/journal.pone.0099727)
Supplement: Table S4 — Carbohydrate and protein levels. The changes in the carbohydrate and protein levels of P. tricornutum cultures were recorded for 10 h after the light quality shift and in the respective BL and RL pre-acclimated reference cultures. (PDF) [file pone.0099727.s004.pdf]

Table S4: Carbohydrate and protein levels. The changes in the carbohydrate and protein levels of *P. tricornutum* cultures were recorded for 10 h after the light quality shift and in the respective BL and RL pre-acclimated reference cultures.

**Time after t_0 (h)**	**0**	**2**	**4**	**6**	**8**	**10**
**BL (pre-acclimated)**						
Carbohydrates [%]	100	100 ± 9	100 ± 6	103 ± 8	100 ± 2	117 ± 7
Proteins [%]	100	100 ± 3	101 ± 3	99 ± 2	98 ± 1	93 ± 1
**RL (pre-acclimated)**						
Carbohydrates [%]	100	102 ± 6	103 ± 8	112 ± 8	112 ± 6	113 ± 5
Proteins [%]	100	100 ± 2	98 ± 3	92 ± 2	90 ± 2	89 ± 2
**RL to BL Shift**						
Carbohydrates [%]	100	96 ± 6	94 ± 8	94 ± 9	96 ± 7	97 ± 7
Proteins [%]	100	103 ± 3	105 ± 4	105 ± 3	103 ± 3	102 ± 2
**BL to RL Shift**						
Carbohydrates [%]	100	108 ± 5	112 ± 5	121 ± 6	125 ± 7	129 ± 9
Proteins [%]	100	96 ± 1	94 ± 1	90 ± 3	86 ± 2	85 ± 2
