# Supplementary material for: The Acclimation of Phaeodactylum tricornutum to Blue and Red Light Does Not Influence the Photosynthetic Light Reaction but Strongly Disturbs the Carbon Allocation Pattern
Source: PLoS One. 2014 Aug 11;9(8):e99727. doi: 10.1371/journal.pone.0099727 (PMC4128583; doi:10.1371/journal.pone.0099727)
Supplement: Table S5 — Carbon partitioning. The relative partitioning of C (carbon) into carbohydrates, proteins and lipids was calculated for the 2 h following the light quality changes. (PDF) [file pone.0099727.s005.pdf]

Table S5: Carbon partitioning. The relative partitioning of C (carbon) into carbohydrates, proteins and lipids was calculated for the 2 h following the light quality changes.

Time after t_0 (h)	RL to BL shift	BL to RL shift
Carbohydrates [%]	-11 ± 52	106 ± 22
Proteins [%]	75 ± 36	16 ± 6
Lipids	36 ± 54	-23 ± 22
