# Supplementary material for: The Acclimation of Phaeodactylum tricornutum to Blue and Red Light Does Not Influence the Photosynthetic Light Reaction but Strongly Disturbs the Carbon Allocation Pattern
Source: PLoS One. 2014 Aug 11;9(8):e99727. doi: 10.1371/journal.pone.0099727 (PMC4128583; doi:10.1371/journal.pone.0099727)
Supplement: Table S6 — Relative metabolite concentrations in BL and RL pre-acclimated P. tricurnutum cultures (in relative counts * cell−1). Significant differences were calculated by a Student's t-test. (PDF) [file pone.0099727.s006.pdf]

Table S6: Relative metabolite concentrations in BL and RL pre-acclimated *P. tricurnutum* cultures (in relative counts * cell^{-1}). Significant differences were calculated by a Student's t-test.

	BL	RL	Significance
Erythrose-4P	0.43 ± 0.35	0.56 ± 0.35	
Sedoheptulose-7P	1.44 ± 0.24	5.55 ± 1.83	
Xylulose-5P	0.99 ± 0.43	1.08 ± 0.15	
Ribose-5P + Ribulose-5P	1.46 ± 0.90	1.20 ± 0.21	
Glucose	15.60 ± 10.69	12.01 ± 1.58	
Glucose-1P	8.85 ± 10.94	2.31 ± 0.83	
Glucose-6P	105.44 ± 51.48	154.62 ± 49.96	
Fructose-6P	25.31 ± 12.28	55.96 ± 14.13	*
PEP	8.37 ± 2.96	10.78 ± 2.96	
Pyruvate	9.85 ± 3.91	6.99 ± 2.20	
Citrate	259.16 ± 271.93	629.31 ± 214.98	
Isocitrate	2.20 ± 1.22	1.95 ± 0.84	
2-Oxoglutarate	12.00 ± 6.19	14.35 ± 3.30	
Succinate	52.90 ± 25.43	40.24 ± 10.21	
Malate	21.57 ± 10.66	19.50 ± 6.54	
Hydropyruvate	78.80 ± 47.58	46.20 ± 9.69	
Tartrate	6.53 ± 3.23	4.56 ± 0.90	
Lactate	339.80 ± 199.13	291.29 ± 115.05	
Succinate-semialdehyde	0.87 ± 0.51	1.06 ± 0.24	
Trehalose-6P	2.44 ± 1.52	2.56 ± 0.35	
UDP	1.23 ± 0.94	0.89 ± 0.40	
UTP	1.97 ± 1.69	1.00 ± 0.59	
