# Supplementary material for: The Acclimation of Phaeodactylum tricornutum to Blue and Red Light Does Not Influence the Photosynthetic Light Reaction but Strongly Disturbs the Carbon Allocation Pattern
Source: PLoS One. 2014 Aug 11;9(8):e99727. doi: 10.1371/journal.pone.0099727 (PMC4128583; doi:10.1371/journal.pone.0099727)
Supplement: Table S7 — Amino acid concentrations in BL and RL pre-acclimated P. tricurnutum cultures (in 10−3 pmol * cell−1). Significant differences were calculated by a Student's t-test. (PDF) [file pone.0099727.s007.pdf]

Table S7: Amino acid concentrations in BL and RL pre-acclimated *P. tricurnutum* cultures (in 10^{-3} pmol * $cell^{-1}$). Significant differences were calculated by a Student's t-test.

	BL	RL	Significance
Glutamine	1.285 ± 0.518	1.229 ± 0.099	
Tryptophan	0.197 ± 0.064	0.143 ± 0.009	
Tyrosine	0.243 ± 0.109	0.180 ± 0.021	
Phenylalanine	0.087 ± 0.036	0.059 ± 0.003	
Serine	0.989 ± 0.389	0.604 ± 0.066	
Methionine	0.097 ± 0.042	0.069 ± 0.006	
Glycine	0.784 ± 0.291	0.493 ± 0.023	
Threonine	0.329 ± 0.139	0.223 ± 0.020	
Valine	0.224 ± 0.058	0.161 ± 0.010	
Leucine + Isoleucine	0.190 ± 0.071	0.123 ± 0.011	
Arginine	0.285 ± 0.071	0.241 ± 0.045	
Ornithine	0.324 ± 0.129	0.366 ± 0.006	
Proline	0.253 ± 0.025	0.228 ± 0.023	
Histidine	0.232 ± 0.040	0.127 ± 0.003	*
