# Supplementary material for: The Acclimation of Phaeodactylum tricornutum to Blue and Red Light Does Not Influence the Photosynthetic Light Reaction but Strongly Disturbs the Carbon Allocation Pattern
Source: PLoS One. 2014 Aug 11;9(8):e99727. doi: 10.1371/journal.pone.0099727 (PMC4128583; doi:10.1371/journal.pone.0099727)
Supplement: File S1 — The calculation of net C partitioning into biomass for carbohydrates, proteins and lipids. A calculation example is given at the right hand side. (PDF) [file pone.0099727.s008.pdf]

File S8: The calculation of net C partitioning into biomass for carbohydrates, proteins and lipids. A calculation example is given at the right hand side.

Data from the FTIR analysis were used to calculate the macromolecular composition of the cells for t_0 (where $CH_{t0[\%]}$ is the carbohydrate, $P_{t0[\%]}$ the protein and $L_{t0[\%]}$ the lipid content at t_0 in % of dry matter) and t_2 (where $CH_{t2[\%]}$ is the carbohydrate, $P_{t2[\%]}$ the protein and $L_{t2[\%]}$ the lipid content at t_2 in % of dry matter). The dry matter per volume culture (where DM_{t0} is the dry matter content at t_0 per litre) is also known from the experiments.

given are:

$$CH_{t0[\%]} = 37\ \%;\ P_{t0[\%]} = 47\ \%;\ L_{t0[\%]} = 17\ \%$$

$$CH_{t2[\%]} = 34\ \%;\ P_{t2[\%]} = 48\ \%;\ L_{t2[\%]} = 17\ \%$$

$$DM_{t0} = 80\ \frac{mg}{l}$$

The dry matter can be used to calculate the amount of the macromolecules per litre at t_0:

[1] $CH_{t0} = CH_{t0[\%]} * DM_{t0}$

$$CH_{t0} = 29.7\ \frac{mg}{l}$$

[2] $P_{t0} = P_{t0[\%]} * DM_{t0}$

$$P_{t0} = 37.5\ \frac{mg}{l}$$

[3] $L_{t0} = L_{t0[\%]} * DM_{t0}$

$$L_{t0} = 13.3\ \frac{mg}{l}$$

where CH_{t0} is the carbohydrate content, P_{t0} the protein content and L_{t0} the lipid content per volume culture at t_0.

Accordingly, the dry matter composition at t_2 is:

[4] $CH_{t2} = CH_{t2[\%]} * DM_{t2}$

$$CH_{t2} = 34\ \%\ DM_{t2}$$

[5] $P_{t2} = P_{t2[\%]} * DM_{t2}$

$$P_{t2} = 48\ \%\ DM_{t2}$$

[6] $L_{t2} = L_{t2[\%]} * DM_{t2}$

$$L_{t2} = 17\ \%\ DM_{t2}$$

where CH_{t2} is the carbohydrate content, P_{t2} the protein content and L_{t2} the lipid content per volume culture at t_2.

The dry matter at t_2 can be calculated as:

[7] $DM_{t2} = DM_{t0} + DM_{\Delta t}$

$$DM_{t2} = 80\ \frac{mg}{l} + DM_{\Delta}$$

where DM_{t2} is the dry matter at t_2 and $DM_{\Delta t}$ is the newly synthesized dry matter between t_0 and t_2. Both are given per volume culture.

Accordingly, the amount of newly synthesized macromolecules can be calculated as:

[8] $CH_{\Delta} = CH_{t2} - CH_{t0}$

$$CH_{\Delta} = 34\ \%\ DM_{t2} - 29.7\ \frac{mg}{l}$$

[9] $P_{\Delta} = P_{t2} - P_{t0}$

$$P_{\Delta} = 48\ \%\ DM_{t2} - 37.5\ \frac{mg}{l}$$

[10] $L_{\Delta} = L_{t2} - L_{t0}$

$$L_{\Delta} = 17\ \%\ DM_{t2} - 13.3\ \frac{mg}{l}$$

where the newly synthesized macromolecules are $CH_{\Delta t}$ (carbohydrates), P_{Δ} (proteins) and $L_{\Delta t}$ (lipids) and are given per volume culture.

In Kroon and Thoms (2006), the amount of carbon (C) per dry matter for the macromolecules is given as 44.4 % [dry matter^{-1}] for carbohydrates, 51.3 % [dry matter^{-1}] for proteins and 69.1 % [dry matter^{-1}] for lipids. Therefore, the amount of net incorporated C between t_0 and t_2 for each macromolecule is:

[11] $C_{CH\Delta t} = 44.4\ \% * CH_{\Delta t}$

$$C_{CH\Delta} = 44.4\ \% * (34\ \%\ DM_{t2} - 29.7\ \frac{mg}{l})$$

[12] $C_{P\Delta t} = 51.3\ \% * P_{\Delta t}$

$$C_{P\Delta} = 51.3\ \% * (48\ \%\ DM_{t2} - 37.5\ \frac{mg}{l})$$

[13] $C_{L\Delta t} = 69.1\ \% * L_{\Delta t}$

$$C_{L\Delta} = 69.1\ \% * (17\ \%\ DM_{t2} - 13.3\ \frac{mg}{l})$$

where the newly incoporated C is given per volume culture for carbohydrates as $C_{CH\Delta t}$, for proteins as $C_{P\Delta t}$ and for lipids as $C_{L\Delta t}$.

Kroon and Thoms (2006) also specify the amount of electrons needed for C for cultures grown with NO_3^- as 4 mol mol^{-1} for carbohydrates, 6.45 mol mol^{-1} for proteins and 5.39 mol mol^{-1} for lipids. The molecular weight for C is 12.011 g mol^{-1}. Therefore, the amount of net absorbed electrons between t_0 and t_2 for each macromolecule is:

[14] $$e_{CH\Delta t} = \frac{C_{CH\Delta}}{12.011 \frac{g}{mol}} * 4 \frac{mol}{mol}$$

$$e_{CH\Delta t} = \frac{44.4\ \% * 4 \frac{mol}{mol}}{12.011 \frac{g}{mol}} * (34\ \%\ DM_{t2} - 29.7 \frac{mg}{l})$$

[15] $$e_{P\Delta t} = \frac{C_{P\Delta}}{12.011 \frac{g}{mol}} * 6.45 \frac{mol}{mol}$$

$$e_{P\Delta t} = \frac{51.3\ \% * 6.45 \frac{mol}{mol}}{12.011 \frac{g}{mol}} * (48\ \%\ DM_{t2} - 37.5 \frac{mg}{l})$$

[16] $$e_{L\Delta t} = \frac{C_{L\Delta}}{12.011 \frac{g}{mol}} * 5.39 \frac{mol}{mol}$$

$$eL_{\Delta t} = \frac{69.1\ \% * 5.39 \frac{mol}{mol}}{12.011 \frac{g}{mol}} * (17\ \%\ DM_{t2} - 13.3 \frac{mg}{l})$$

where the amount of electrons used for net accumulation of carbohydrates is $e_{CH\Delta t}$, for net accumulation of proteins is $e_{P\Delta t}$ and for net accumulation of lipids is $e_{L\Delta t}$.

The net amount of absorbed irradiance $e_{all\Delta}$ can be calculated from the net oxygen evolution at growth light, the Chlorophyll a content per volume culture, the amount of electrons needed for the evolution of one O_2 the time between t_0 and t_2 (2 hours).

$$e_{all\Delta t} = 36 \frac{\mu mol\ O_2}{mg\ Chl\ a * l * h} * 2.3\ mg\ Chl\ a * 4 \frac{mol\ electrons}{mol\ O_2} * 2h$$

$$e_{all\Delta t} = 0.7 \frac{mmol\ electrons}{l}$$

The overall absorbed electrons can also be calculated as:

[17] $$e_{all\Delta t} = e_{CH\Delta t} + e_{P\Delta t} + e_{L\Delta t}$$

Therefore, $e_{all\Delta t}$ is also:

$$[18]\ e_{all\Delta t} = \frac{44.4\ \% * 4\ \frac{mol}{mol}}{12.011\ \frac{g}{mol}} * (CH_{t2[\%]} * DM_{t2} - CH_{t0[\%]} * DM_{t0}) +$$

$$\frac{51.3\ \% * 6.45\ \frac{mol}{mol}}{12.011\ \frac{g}{mol}} * (P_{t2[\%]} * DM_{t2} - P_{t0[\%]} * DM_{t0}) +$$

$$\frac{69.1\ \% * 5.39\ \frac{mol}{mol}}{12.011\ \frac{g}{mol}} * (L_{t2[\%]} * DM_{t2} - L_{t0[\%]} * DM_{t0})$$

As every value except DM_{t2} is known, formula [18] can be used for the calculation of the dry matter at t_2.

$$DM_{t2} = 82 \frac{mg}{l}$$

From here, the calculation of net incorporated C into carbohydrates, proteins and lipids (formula [11-13]) is easily possible.
